# Children with Specific Language Impairment are not impaired in the acquisition and retention of Pavlovian delay and trace conditioning of the eyeblink response^☆^

**DOI:** 10.1016/j.bandl.2013.08.001

**Published:** 2013-12

**Authors:** Mervyn J. Hardiman, Hsin-jen Hsu, Dorothy V.M. Bishop

**Affiliations:** a Department of Experimental Psychology, University of Oxford, South Parks Road, Oxford, OX1 3UD, United Kingdom; b National Kaohsiung Normal University, Taiwan

**Keywords:** Specific Language Impairment, SLI, Pavlovian, Classical, Conditioning, Eyeblink, Delay, Trace, Cerebellum, Procedural

## Abstract

Three converging lines of evidence have suggested that cerebellar abnormality is implicated in developmental language and literacy problems. First, some brain imaging studies have linked abnormalities in cerebellar grey matter to dyslexia and specific language impairment (SLI). Second, theoretical accounts of both dyslexia and SLI have postulated impairments of procedural learning and automatisation of skills, functions that are known to be mediated by the cerebellum. Third, motor learning has been shown to be abnormal in some studies of both disorders. We assessed the integrity of face related regions of the cerebellum using Pavlovian eyeblink conditioning in 7–11 year-old children with SLI. We found no relationship between oral language skills or literacy skills with either delay or trace conditioning in the children. We conclude that this elementary form of associative learning is intact in children with impaired language or literacy development.

## Highlights

• We tested the integrity of small regions of the cerebellum and hippocampal formation in children with SLI.
• Pavlovian delay conditioning engages elements of the procedural memory system.
• Pavlovian trace conditioning engages procedural and declarative memory systems.
• Children with Specific Language Impairment learned to delay and trace conditioning.
• Brain regions engaged in eyeblink conditioning are normal in children with SLI.

## 1 Introduction

Several theories have been proposed to explain why some children have a selective problem in language acquisition, a condition known as specific language impairment (SLI). The language difficulties, which typically are most apparent in learning language structure – i.e. phonology and syntax – have been variously attributed to problems with auditory perception, impairments of short-term memory, or inadequate development of a specialised language module (Bishop, 1992). More recently, however, another account has achieved prominence, namely the idea that SLI reflects impaired procedural learning.

A procedural learning account of a developmental disorder was first suggested by Nicolson and Fawcett (1990) in their automaticity account of dyslexia. The authors argued that slow and laborious reading together with impairments in motor skills were symptomatic of problems in “proceduralisation” of learned knowledge. Nicolson (2001) provided a neural locus for such a procedural deficit by attributing problems with processing speed, phonological short-term memory and motor skills to a general deficiency in cerebellar function. In subsequent writings, Nicolson and Fawcett (2007, 2011) extended their theory to cover other developmental disorders, including SLI, by drawing upon the ideas of Ullman and Pierpont (2005) expounded in the Procedural Deficit Hypothesis of SLI. Nicolson and Fawcett proposed that both dyslexia and SLI were caused by impairments of procedural memory systems specialised for language. Dyslexia involved disruption of the cortico-cerebellar pathway but SLI involved disruption of the cortico-striatal pathway.

Ullman and Pierpont (2005) had argued that the primary difficulties in SLI must lie within the cortico-striatal pathways because children with SLI are particularly poor at learning grammatical forms, but have relatively unimpaired lexical knowledge. Their reasoning was based upon the widely-used distinction between procedural and declarative memory systems which mapped onto different aspects of language. Rule-based learning of grammar relied upon the cortico-striatal part of the procedural memory system whereas lexical learning, through the association of sound and meaning, relied upon the declarative memory system comprising the hippocampus and associated neocortex (Ullman, 2001, 2004). Ullman and Pierpont (2005) emphasised the role of the caudate nucleus and its connections with BA44 and 45. But they also cited difficulties in accessing lexical memories, often seen in SLI and dyslexia, as evidence of impairment in both the basal ganglia and cerebellum. In a similar vein Nicolson and Fawcett (2007) argued that deficiencies in both cortico-striatal and cortico-cerebellar pathways accounted for the frequent comorbidities between SLI, dyslexia and motor impairment (Bishop & Snowling, 2004).

The theoretical account of SLI described above has received some experimental support. A deficit in procedural memory related to grammatical difficulties has been shown in children with SLI (Hedenius et al., 2011; Tomblin, Mainela-Arnold and Zhang, 2007; but see Gabriel, Maillart, Guillaume, Stefaniac, & Meulemans, 2011). However, other reports provide evidence that both procedural and declarative memory systems are compromised in SLI (Lum, Conti-Ramsden, Page & Ullman, 2012; Lum, Gelgec, & Conti-Ramsden, 2010).

### 1.1 Cerebellum and language impairment

The cerebellum has featured more prominently in causal accounts of dyslexia rather than SLI. Several lines of evidence now suggest that a cerebellar deficit is implicated in SLI. *First*, there is considerable overlap between the two disorders (Bishop & Snowling, 2004). In particular many children with SLI are poor readers (McArthur, Hogben, Edwards, Heath, & Mengler, 2000). *Second*, volumetric analyses have revealed abnormalities in size and symmetry within the right anterior lobe in children and adults with dyslexia (Eckert et al., 2003; Leonard and Eckert, 2008), but in children with SLI, similar deficits have been found in cerebellar lobules VIIIA and right Crus I (Hodge et al., 2010). These abnormalities correlate with similar irregularities in left Heschl’s gyrus, planum temporale and inferior frontal cortex. *Third*, as well as problems with rule-based grammatical learning, children with SLI frequently have marked deficiencies in phonological memory (Graf Estes, Evans, Alibali & Saffran, 2007).

A growing body of evidence suggests that parts of the cerebellum, together with BA44 and 45, are engaged in phonological memory. Lobules HVI, Crus I, VIIB and VIII are active during verbal and non-verbal working memory tests (Desmond, Gabrieli, Wagner, Ginier, & Glover, 1997; Durisko & Fiez, 2010; Küper et al., 2011; Thürling et al., 2012). A comprehensive explanation was put forward by Desmond and colleagues. Throughout a series of functional MRI studies the authors argued that right cerebellar lobules HVI and Crus I in particular are part of the circuit engaged in articulatory rehearsal, which also involves BA44/45, lateral BA6 (pre-motor) and medial BA6 (supplementary motor cortex). In addition they proposed that cerebellar lobule VIIB together with BA40 (left inferior parietal and supramarginal gyrus) is involved in phonological memory storage and retrieval (Chein & Fiez, 2001; Chen & Desmond, 2005). Thus their work makes a clear link between the cerebellum and the comparison of phonological and articulatory information leading to the adjustment of content in short term memory as part of the phonological memory system described by Baddeley (1992).

Whether cerebellar activity is critical simply for the control the articulatory muscles (Riecker et al., 2005), or for articulatory rehearsal mechanisms or other aspects of language remains largely unknown. Some evidence for a fundamental role in language comes from cerebellar patients who have presented with poor working memory and language (Cooper et al., 2012; Justus, Ravizzi, Fiez, & Ivry, 2005; Oki, Takahashi, Miyamoto, & Tachibana, 1999; Riva & Giorgi, 2000; Silveri, Leggio, & Molinari, 1994; Stoodley & Schmahmann, 2009b) including agrammatic speech (Silveri, Di Betta, Filippini, Leggio, & Molinari, 1998) and poor verbal fluency (Richter et al., 2007). Nevertheless not all cerebellar patients have difficulties with language (Alexander, Gillingham, Schweitzer, & Stuss, 2012; Frank et al., 2007; Helmuth, Ivry, & Shimizu, 1997; Richter et al., 2004, 2007).

### 1.2 Eyeblink conditioning

To date, most studies that have tested the Procedural Deficit Hypothesis in SLI have used a version of the serial reaction time task to explore procedural memory. The striatum has been shown to be essential for long-term retention of this type of sequence motor learning (Seger & Cincotta, 2002), but the cerebellum is important during initial learning stages (Doyon et al., 2009). In contrast we tested the generality of a procedural and/or declarative deficit in SLI and examined further the neurobiological basis of such a deficit by the use of a simple form of associative learning, Pavlovian conditioning of the eyeblink response. Pavlovian delay conditioning is dependent upon the cerebellum and has been shown to be impaired in children with dyslexia (Coffin, Baroody, Schneider, & O’Neill, 2005) and adults (Nicolson, Daum, Schugens, Fawcett, & Schulz, 2002). We argue that children with SLI or at least those who are poor at reading will also show impairment in eyeblink conditioning.

The eyeblink response may be conditioned by presenting a stimulus, such as a tone, that initially does not elicit an eyeblink (the conditioned stimulus, CS), closely followed by a brief puff of air to the eye (the unconditioned stimulus, US) which will always cause a blink (the unconditioned response, UR). After several presentations of the paired CS and US a conditioned response (CR) will appear that both precedes the US and is maximal about the time of the maximum UR. In delay conditioning the US co-terminates with the CS and in trace conditioning a delay is inserted between the offset of the CS and the onset of the US such that a memory trace of the CS had to be formed to allow conditioning to proceed.

Clark and Squire championed the idea that delay conditioning is an example of non-declarative learning whereas trace conditioning is an example of declarative learning (Clark and Squire, 1998; Clark, 2011). Delay conditioning occurs without knowledge of the relationship between the CS and US whereas trace conditioning requires knowledge of the relationship between the CS and US. This account of delay and trace conditioning is not universally accepted: Lovibond, Liu, Weidemann, and Mitchell (2011) suggested that both depend upon a single declarative memory system. Whatever the overall mechanism we can use differences in the degree of engagement of elements within the language system by delay and trace conditioning, to test the idea that these parts may be impaired differentially in children with SLI as suggested by Ullman and Pierpont (2005).

### 1.3 Cerebellum and conditioning

Delay conditioning is dependent upon the cerebellum. Lobules HVI and Crus I ipsilateral to the US are essential for normal delay conditioning in rabbits (Attwell, Ivarsson, Millar, & Yeo, 2002; Clark, Zhang, & Lavond, 1997; Hardiman & Yeo, 1992; Yeo, Hardiman, & Glickstein, 1985a) and man (Daum et al., 1993; Gerwig et al., 2003, 2010;). Lobules HVI and Crus I contain microzones determined by somatosensory information from the face via the inferior olive (Dimitrova et al., 2002; Hesslow, 1994; Mostofi, Holtzman, Grout, Yeo, & Edgley, 2010; Van Ham & Yeo, 1992), where information related to the CS and US come together (physiology: Berthier & Moore, 1986; Hesslow, 1994; McCormick & Thompson, 1984; Schreurs, Gusev, Tomsic, Alkon, & Shi, 1998; anatomy: Rosenfield & Moore, 1995; Yeo, Hardiman, & Glickstein, 1985b). Although the precise role of the cerebellar cortex in conditioning remains controversial (Christian & Thompson, 2003) face related cortical microzones influence sites within the deep cerebellar nuclei that are essential for both delay and trace conditioning (McCormick, Clark, Lavond & Thompson, 1982; Woodruff-Pak, Lavond, & Thompson, 1985).

Trace conditioning is more complicated. The simplest explanation is that the period between the offset of the CS and onset of US is bridged by neuronal circuitry that retains a memory of the CS. The formation of such a memory may be dependent upon the hippocampus (Kryukov, 2012). Alternatively a memory of the CS may be maintained elsewhere and the hippocampus is essential for short term memory of the CS/US association. Lesions in man and non-human animals have shown that, in addition to the cerebellum, the hippocampus and associated structures are essential for the initial stages of trace conditioning (McGlinchey-Berroth, Carillo, Gabrieli, Brawn, & Disterhoft, 1997; Moyer, Deyo, & Disterhoft, 1990; Weible, McEchron, & Disterhoft, 2000). The involvement of the hippocampus is transient (Kim, Clark, & Thompson, 1995; Takehara-Nishiuchi & McNaughton, 2008), permanent storage of the trace may engage medial prefrontal cortex (McLaughlin, Skaggs, Churchwell, & Powell, 2002; Siegel, Kalmbach, Chitwood, & Mauk, 2012) and dorsolateral prefrontal cortex (Weiss & Disterhoft, 2011).

### 1.4 Cerebellum, language and laterality

Lobules HVI and Crus I, the same regions of the cerebellar cortex that are active during Pavlovian conditioning of the eyeblink response (Cheng, Disterhoft, Power, Ellis, & Desmond, 2008; Ramnani, Toni, Josephs, Ashburner, & Passingham, 2000) are also active during fMRI experiments when overt and covert speech is engaged (Ackermann, Mathiak, & Riecker, 2007). Activity is restricted to a subset of cerebellar lobules because only they are engaged in fine control of the articulatory muscles required for fluent speech (Dhanjal, Handunnetthi, Patel, & Wise, 2008). Simple movement of the tongue, jaw and lips, as well as vowel production, elicit bilateral activation within HVI (Grabski et al., 2011) and a smaller activation within HVIII (Corfield et al., 1999; Grodd, Hülsmann, Lotze, Wildgruber, & Erb, 2001). The right cerebellum in particular is active in language (Stoodley & Schmahmann, 2009a) because regions in control of the articulatory muscles are influenced by and/or have some influence over neurons within left BA6, 44, 45 (Berl et al., 2012; Jansen et al., 2005; Lidzba, Schwilling, Grodd, Krägeloh-Mann, & Wilke, 2011; Lidzba, Wilke, Staudt, Krägeloh-Mann, & Grodd, 2008; Wilke et al., 2006) that also control orofacial musculature (Kelly et al., 2010).

### 1.5 Eyeblink conditioning in SLI

Steinmetz and Rice (2010) examined the role of the cerebellum using Pavlovian delay conditioning of the eyeblink response in children and young adults with SLI. Participants were conditioned by applying the unconditioned stimulus to the left eye thereby engaging the left cerebellum and associated structures. The authors found no difference in conditioning compared with typically developing children.

However, as reviewed above, there is considerable evidence to suggest that it is regions within the right cerebellum that are more engaged than the left in language. Training with the unconditioned stimulus applied to the left eye would have engaged mainly the left cerebellum (Disterhoft, Kwan, & Warren, 1977; Gruart & Yeo, 1995; McCormick & Thompson, 1984; Miller et al., 2003). In addition eyeblinks were recorded from the left eye and would not have tested the integrity of the right cerebellum. Thus the study by Steinmetz and Rice (2010) did not provide a conclusive test of the hypothesis of a cerebellar deficit underlying phonological memory and procedural learning in SLI.

### 1.6 The current study

We examined conditioning in four groups of children: 7–11 year old children with SLI, subdivided into those with and without reading difficulties, children matched in age with the SLI group, and younger children matched in language level with the SLI group. Language (Rice, Wexler, & Hershberger, 1998) and motor (Bishop & Edmundson, 1987) development in children with SLI is delayed by several years. We included a younger typically-developing group of children to test whether any deficit in conditioning could be due to a simple delay in development of the cerebellum. If conditioning is worse in younger than older typically developing children, and children with SLI perform the same as younger children, the likely cause will be a delay in development of the cerebellum. However, if children with SLI perform worse than language matched, younger typical children we can conclude that there is a more substantial impairment of cerebellum function.

Children were given a comprehensive assessment of language and literacy, allowing us to consider how eyeblink conditioning related to language ability, and whether deficits in conditioning characterised just those with literacy problems. We extended previous studies by examining delay and trace conditioning in the same participants thus allowing us to test the procedural deficit hypothesis which suggests that children with SLI will have poor procedural learning but relatively intact declarative memories.

### 1.7 Predictions

A. Children with SLI who have evidence of impairment in procedural learning i.e. poor grammar, will fail to learn *delay* conditioning. In addition, we anticipated that the extent of impairment in delay conditioning would be associated with phonological short-term memory.
B. Children with SLI who have evidence of weak declarative learning, i.e. poor expressive vocabulary, will be impaired in *trace* conditioning but show unimpaired delay conditioning. As noted above both forms of conditioning involve the cerebellum, but trace conditioning also involves cortical mechanisms. Differential impairment of trace conditioning in those with weak vocabulary would be indicative of impaired cortical mechanisms.
C. A deficit in both delay and trace conditioning will not allow a distinction between a deficit in procedural and declarative memory systems but will provide evidence for a cerebellar deficit.
D. If SLI involves a maturational lag in cerebellar development then we would expect children with SLI to show less conditioning than age matched controls, but similar levels of conditioning to younger children of a similar language level.
E. A final possible outcome is that only those children with SLI who are poor readers will fail to condition. This would be consistent with results from (Coffin, Baroody, Schneider, & O’Neill, 2005) and would fit with the proposal by Nicolson and Fawcett (2011) which regards cerebellar impairment as a correlate of reading difficulties.

## 2 Methods

### 2.1 Participants

The participants were a subset of participants in a language training study described by Hsu and Bishop (2013). A breakdown of the number of children and adults participating in the study is given in Table 1. There were four groups of children: two groups of typically developing children were matched in age (AM: *n* = 21) or raw language scores (LM: *n* = 21) to two groups of children with SLI. Children with SLI were identified by assessment on six tests assessing phonological processing, receptive and expressive vocabulary, receptive grammar, syntactic formulation and comprehension. Children were placed in the SLI group if their score was more than one standard deviation below the norm (85) on two or more of the tests. The children with SLI were divided into two groups according to their reading ability measured by the Test Of Word Reading Efficiency subtests of sight word reading efficiency (word reading) and phonemic decoding efficiency (non-word reading). Children who obtained a score more than one standard deviation below the norm on both tests were placed in the poor reader group (SLI_PR: *n* = 22) and the remainder in the typical reader group (SLI_TR: *n* = 17).

The LM group was defined as having the same raw score as the SLI groups for receptive and expressive vocabulary, and was on average 2–3 years younger than the SLI groups (see Table 1). In order to predict how well the groups should perform on delay conditioning we investigated their proficiency in grammar. According to the grammar and vocabulary scores and the predictions set out in the introduction the groups should differ in their delay conditioning following the order AM > SLI_TR = LM > SLI_PR. In contrast both the SLI groups together with the LM group should show poor trace conditioning. All participants underwent delay conditioning but some children were unavailable for trace conditioning as shown in Table 1.

The study was approved by the University of Oxford Central University Research Ethics Committee; parents of all participants gave written informed consent, and the children themselves gave assent after the study was explained in age-appropriate language.

### 2.2 Psychometric tests

All children except the AM group underwent the full battery of psychometric tests as detailed in Table 1. The AM group were not tested for their receptive or expressive vocabulary.

### 2.3 Conditioning environment

Conditioning took place in a custom made mobile laboratory. Each participant was fitted with three sintered silver/silver chloride electrodes filled with non-allergenic electrode gel. The positive electrode was placed next to the outer canthus of the right eye. The negative electrode was placed immediately above the positive electrode at an angle of forty-five degrees so that both electrodes sat over the orbicularis oculi muscle. The positioning of the electrodes minimised the recording of activity due to movements of the cheek which was very apparent in small children. A ground electrode was placed on the forehead. A builder’s hard hat fitted with a flexible tube to deliver a puff of air was place on the participant’s head. The flexible tube was placed one centimetre away from the outer canthus of the right eye. Sound attenuating headphones were then fitted over both ears and secured to the hat.

Each participant was instructed to relax and enjoy a DVD played on a 15 in. laptop PC situated one metre away. The DVD sound track was played at a level so that the participant could easily hear it. Pilot testing indicated that without the addition of the sound track children soon lost interest in the film. With the sound track all children remained engaged in the film throughout the experiment.

### 2.4 EMG recording

EMG activity from the right orbicularis oculi was amplified using a Nuamps (Neuroscan) and recorded using SCAN4 software (Neuroscan) with an analogue to digital conversion rate of 1000 Hz and bandpass filter from 2 Hz to 300 Hz.

Stimulus delivery was controlled by a program developed in Builder C++ (Embarcadero) and stimulus timings checked by oscilloscope. Intensity of the tone and white noise were regularly checked using an artificial ear (Bruel and Kjaer type 4153) and sound analyser (Bruel and Kjaer type 2260).

### 2.5 Training

#### 2.5.1 Pseudoconditioning

The first twenty trials before delay conditioning consisted of ten CS and ten US trials presented randomly with a minimum time delay between the stimuli of 600 ms so that the stimuli never overlapped. The CS and US parameters are described below.

#### 2.5.2 Conditioning

Delay conditioning consisted of 100 trials of which eighty trials were paired, ten trials were CS alone and five trials were US alone. Every tenth trial starting at trial five was a CS alone trial. Every twentieth trial starting from trial 10 was a US alone trial. The CS was a 1 kHz tone, duration 500 ms, intensity 86 dB SPL, delivered via Sennheiser HD25 SII headphones to the right ear only. The US was a puff of medical air (BOC), duration 100 ms, intensity 1–5 psi at source, directed to the outer canthus of the right eye. We adjusted the intensity of the airpuff for each participant to elicit an eyeblink that matched the natural blink. This was particularly important in children as some were very sensitive to the airpuff. The interval between the onset of the CS and the onset of the US (inter-stimulus interval, ISI) was 400 ms. The inter-trial interval was selected at random between 20 and 30 s.

Trace conditioning was conducted seven days after delay conditioning. Trace conditioning was identical to delay conditioning except the ISI was 1000 ms making a trace interval of 500 ms. We deliberately kept the CS duration the same as in the delay condition but altered the ISI between delay and trace. Thereby we could readily discern whether there was generalisation between the CSs used in the two sessions as has been shown in children (Jacobsen et al., 2012).

### 2.6 Eyeblink analysis

The Neuroscan data was analysed using a program developed in Matlab (Mathworks) using several functions from within the EEGLAB (Delorme & Makeig, 2004) environment. The raw EMG was epoched, rectified, low pass filtered at 20 Hz and the baseline removed. Trials were rejected if they had eyeblinks in the baseline period, responses immediately after the onset of the CS (see below for Alpha response), large eye opening in CS period or the absence of a UR on a paired trial.

The baseline period was 300 ms before CS onset. An eyeblink in the baseline period was defined as a response greater than 5 standard deviations above baseline for 25 ms or a response with a maximum amplitude of 10 standard deviations above baseline. An Alpha response was defined as a reflex response to the onset of the CS greater than the baseline amplitude +5 standard deviations occurring between 1 and 99 ms after the onset of the CS. For delay conditioning a CR was defined as a response greater than baseline +5 standard deviations, occurring between 100 ms after the onset of the CS, to the start of the US, a period of 300 ms. The value selected resulted in a criterion close to 1 mm of movement for each group: MN(SD): AM = 17.82(10.07); LM: 14.73(9.83); SLI_GR: 15.78(10.4); SLI_PR: 15.15(8.94); where 1 mm ≈ 15 μV.

For trace conditioning a CR was defined as a response greater than the baseline +5 standard deviations, occurring between 700 ms after the onset of the CS, to the start of the US, a period of 300 ms. Because generalisation occurred between the CSs used in delay and trace conditioning, initial trials of Session 2 revealed responses within the CS period. We therefore also analysed responses from 100 ms of CS onset until 700 ms in Session 2.

Although we employed CS and US alone trials, as well as trials in which the CS and US were paired, conditioning was most clearly shown by analysis of the paired trials. The data from 85 paired trials were collapsed to remove the last 5 trials, together with 10 CS alone trials and 5 US alone trials thus leaving 8 blocks of 10 paired trials to analyse. We originally analysed the data in blocks of 5 paired trials however there was no meaningful difference between the analysis in blocks of 5 or 10. In order to compare more readily our results with earlier studies we have presented the data in blocks of 10 paired trials.

The development of CRs across trials is an indication of how well the participants learned. Thus for the main analysis we looked at the number of CRs across blocks of ten paired trials to determine if learning differed across groups. CRs were also analysed for the peak amplitude in order to determine whether essential neuronal circuit had been compromised, but not fully incapacitated, leading to a weak response. Initially the CR develops within the US period and as conditioning proceeds it increases in amplitude and duration so that it occurs before the US but is maximal at about the time of the peak UR. We therefore took as a second measure of learning the latency to onset of the CR. Since well-adapted CRs peak just after the US onset and maladaptive CRs peak close to the CS onset we also looked at the latency to the peak amplitude. As an indicator of responsiveness or sensitivity within the eyeblink reflex circuit we analysed the UR peak amplitude and latency to this peak.

Data was analysed for statistical significance at *p* < .05 using GLM Repeated Measures within IBM SPSS version 20. Where the assumption of sphericity was violated degrees of freedom were corrected using Greenhouse-Geisser estimates of sphericity. Dependent variables are given above with between-subjects factor GRP consisting of AM, LM, SLI_TR and SLI_PR. The within-subjects factor was BLK where each of 8 blocks was the mean of ten paired trials for each participant. Relationships between behavioural data and conditioning data were explored using Pearson correlation.

## 2 Results

### 3.1 Psychometric tests

Table 1 confirms that the SLI group had poor grammar. They fell below the norm by 1 SD on TROG. The LM and SLI groups were well matched on receptive and expressive vocabulary. Note that the SLI groups scored within the normal range for receptive vocabulary and below the normal range for expressive vocabulary. The SLI_PR group was selected from those children with SLI who fell below the norm for reading by 1 SD. The SLI_PR group, but not the SLI_TR group, fell 1 SD below the norm on the NEPSY non-word repetition task.

### 3.2 Session 1: delay conditioning

All salient aspects of conditioning were shown by analysis of paired trials. A summary of the statistical analysis for delay conditioning is given in Table 2. Mean and variation for all response parameters are given in Supplementary Table S1. The main result is that there was no significant difference between the typically developing children and children with SLI. No significant difference was found for the main effect of GRP or for the interaction between GRP and BLK for any measure of CR or UR.

Overall there was clear evidence of conditioning. The main effect for BLK was significant for several CR and UR parameters: the number of CRs, their amplitude and the latency to peak amplitude, UR amplitude and UR latency to peak amplitude.

**Fig. 1:**
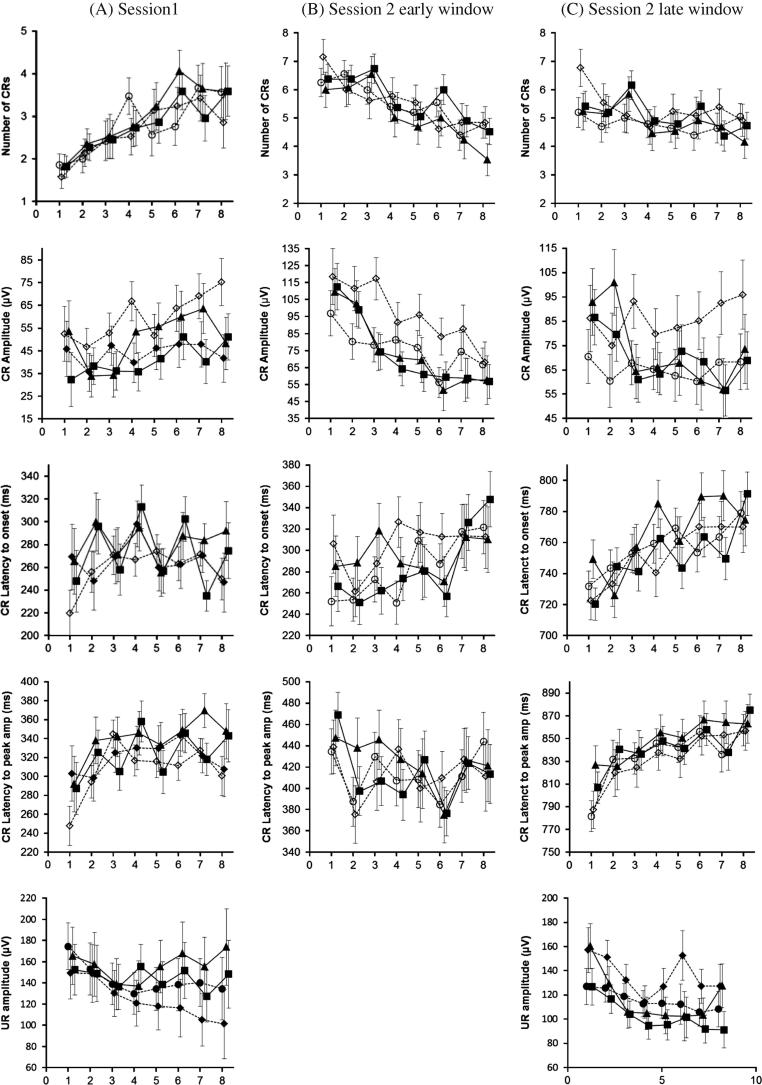
Development of delay and trace conditioning across 80 paired trials. Column A = Delay conditioning. Column B = Trace conditioning early time window. Column C = trace conditioning late time window. Row 1–4 = CR parameters. Row 5 = UR parameters. SLI_TR = solid line with triangles; SLI_PR = solid line with squares. AM = dotted line with circles; LM = dotted line with diamonds. X axis = BLOCK number. Y axis is optimised for each plot. For amplitudes 1 mm ≈ 15 μV. Error bars = SE.

First, as shown in Fig. 1A, row 1, the number of CRs increased significantly as the conditioning session progressed. For the number of CRs, within-subjects contrasts revealed significant differences between blocks 1, 2 and 3 when compared with block 8: BLK 1 v 8, *F*(1, 77) = 29.37, *p* < .001, $\eta_{p}^{2}$ = .28; BLK 2 v 8, *F*(1, 77) = 16.10, *p* < .001, $\eta_{p}^{2}$ = .17; BLK 3 v 8, *F*(1, 77) = 12.05, *p* = .001, $\eta_{p}^{2}$ = .13.

Second, CRs increased slightly in amplitude across blocks (Fig. 1A, row 2) but did not show a strong change in CR timing, either in onset or to the peak amplitude (Fig. 1A, rows 3, 4). For CR amplitude only blocks 2 and 3 differed significantly from block 8: BLK 2 v 8, *F*(1, 77) = 6.93, *p* = .010, $\eta_{p}^{2}$ = .083; BLK 3 v 8, *F*(1, 77) = 5.37, *p* = .023, $\eta_{p}^{2}$ = .065. For CR latency to peak amplitude block 1 differed with block 8, *F*(1, 37) = 5.14, *p* = .029, $\eta_{p}^{2}$ = .122.

### 3.3 Session 2: Generalisation of delay conditioning (early time window)

From the first trial of the second session of conditioning responses were made as if delay conditioning had continued. CRs were made in the same time period as during delay conditioning (Fig. 1A, row 3, block8 vs Fig. 1B, row 3, block 1). We therefore examined all responses in the second session through an early time window calculated to capture these generalised responses. A summary of the ANOVA for generalised delay conditioning is given in Table 2, Early Time Window.

**Fig. 2:**
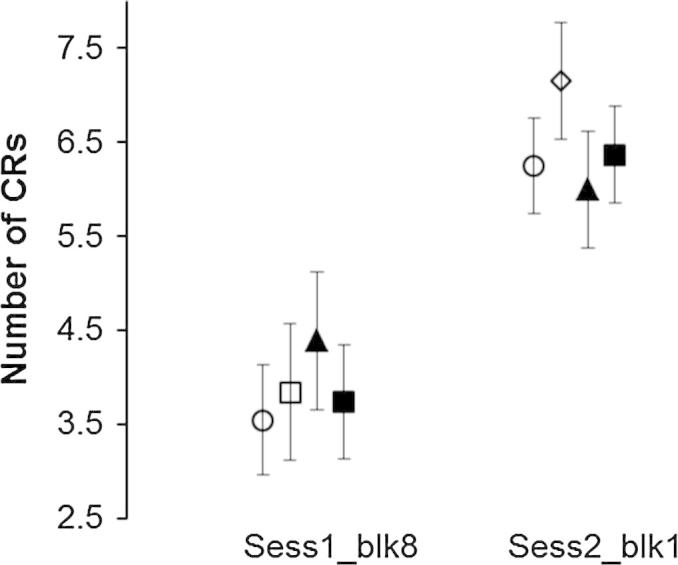
Consolidation and generalisation. Comparison between the number of CRs made on block 8 of Session 1 (delay conditioning) and in the early time window of block 1, Session2 (trace conditioning). SLI_TR = solid line with triangles; SLI_PR = solid line with squares. AM = dotted line with circles; LM = dotted line with diamonds. Error bars = SE.

The effect of consolidation had occurred within the week intervening the sessions of delay and trace. This was seen as the immediate production of CRs during trace conditioning that matched the timing of delay conditioning. This effect was clearly seen in most children but particularly in those whose performance in delay conditioning was below the average. The relationship between sessions 1 and 2 was explored statistically by examining the number of CRs in the final block 8 of paired trials in delay conditioning with responses produced in the early time window of the second session block 1 (see Fig. 2). For this analysis only those subjects who underwent both sessions conditioning were analysed (see Table 1). The main effect of GRP was not significant: *F*(1, 61) = .912, *p* = .044, $\eta_{p}^{2}$ = .043. The main effect of BLK was significant: *F*(1, 61) = 58.01, *p* < .001, $\eta_{p}^{2}$ = .488. There was no interaction between GRP and BLK: *F*(3, 61) = .912, *p* = .44, $\eta_{p}^{2}$ = .043.

Together with Fig. 2 these results show that all groups produced significantly more CRs on the first block of session 2 than in the last block of session 1. Note that differences between values given in Fig. 2 and Fig. 1A (row 1, block 8) are due to the difference in number of participants. Some participants in the delay group did not undergo the second session of conditioning.

The effect of extinction was seen as session 2 proceeded. CRs generalised from delay conditioning diminished in size and number as CRs timed to the parameters of trace conditioning appeared. Thus generalised CRs extinguished as trace conditioning proceeded. The extinction of early CRs related to delay conditioning is shown by an analysis of BLK for the number and amplitude of CRs produced in the early trace time window given in Table 2. Inspection of Fig. 1B, row 1, together with within-subjects contrasts revealed significant decrease in CRs from block 1 to 6: BLK 1 v 8, *F*(1, 61) = 32.63, *p* < .001, $\eta_{p}^{2}$ = .349; BLK 2 v 8, *F*(1, 61) = 35.09, *p* < .001, $\eta_{p}^{2}$ = .365; BLK 3 v 8, *F*(1, 61) = 35.08, *p* < .001, $\eta_{p}^{2}$ = .378; BLK 4 v 8, *F*(1, 61) = 8.65, *p* = .005, $\eta_{p}^{2}$ = .124; BLK 5 v 8, *F*(1, 61) = 6.31, *p* = .015, $\eta_{p}^{2}$ = .094; BLK 6 v 8, *F*(6, 31) = 9.89, *p* = .003, $\eta_{p}^{2}$ = .139. There was a significant effect of BLK for CR amplitude in the early time window (see Table 2). Together with the plot of CR amplitude against trial block in Fig. 1B, row 2, the within-subjects contrasts revealed that CR amplitude decreased significantly across blocks 1–5 compared with block 8: BLK 1 v 8, *F*(1, 61) = 50.45, *p* < .001, $\eta_{p}^{2}$ = .453; BLK 2 v 8, *F*(1, 61) = 41.48, *p* < .001, $\eta_{p}^{2}$ = .405; BLK 3 v 8, *F*(1, 61) = 25.92, *p* < .001, $\eta_{p}^{2}$ = .289; BLK 4 v 8, *F*(1, 61) = 12.62, *p* = .001, $\eta_{p}^{2}$ = .172; BLK 5 v 8, *F*(1, 61) = 6.58, *p* = .013, $\eta_{p}^{2}$ = .097.

**Fig. 3:**
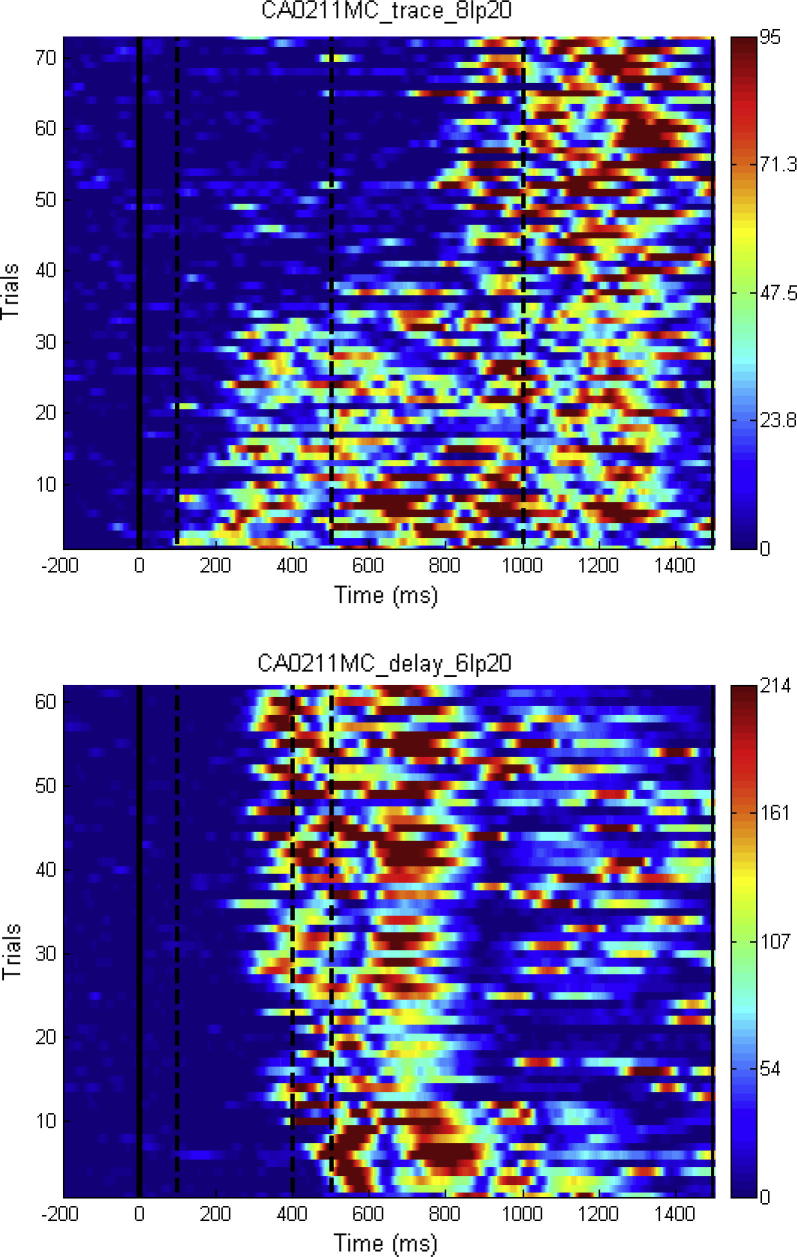
Development of CRs from delay through trace conditioning. Top graph all good trials in trace conditioning. Bottom graph all good trials in delay conditioning. Response amplitude in μV is colour coded. Trial 1 is at the bottom of each graph. This AM participant produced 32 CRs during delay conditioning and one week later produced 32 early CRs and 36 late CRs during trace conditioning.

The pattern of consolidation, generalisation and extinction is illustrated in Fig. 3, a plot of all good trials for each session for one AM participant. Trials are plotted on the *y*-axis starting from trial 1, showing the chronological order from delay through trace conditioning. In the top graph, showing the development of responses to trace conditioning, there is an immediate increase in size of the CR related in time to the delay parameters. Early responses are long then become bimodal and eventually the early responses extinguish to leave CRs well timed to the trace parameters. In some participants the early responses remained through trace conditioning taking the form of an eyeblink extending across the trace period, in other participants the response remained bimodal, the first response timed to the delay parameters and a later one timed to the trace parameters.

### 3.4 Session 2: Trace conditioning (late time window)

Statistical analysis of trace conditioning was done on responses made in the late time window given in Table 2. Analysis of the number of CRs revealed that all groups learned at the same rate and to the same amount. Main effects of GRP and GRP x BLK interaction were non-significant but the main effect of BLK was significant. Within-subjects contrasts revealed significant differences between blocks 1 and 3 and block 8. BLK 1 v 8, *F*(1, 61) = 10.39, *p* < .001, $\eta_{p}^{2}$ = .145; BLK 3 v 8, *F*(1, 61) = 6.99, *p* = .010, $\eta_{p}^{2}$ = .103. By inspection of Fig. 1C, row 1, we can see that the contrasts indicate a significant reduction in CRs.

For CR onset latency the main effect of BLK was significant (see Table 2). By inspection of Fig. 1C, row 3, and by analysis of within-subjects contrasts we can see that there is a significant reduction in latency in blocks 1, 2, 3, 5: BLK 1 v 8, *F*(1, 55) = 27.94, *p* < .001, $\eta_{p}^{2}$ = .337; BLK 2 v 8, *F*(1, 55) = 19.43, *p* < .001, $\eta_{p}^{2}$ = .261; BLK 3 v 8, *F*(1, 55) = 10.42, *p* = .002, *ηp2* = .159; BLK 5 v 8, *F*(1, 55) = 4.68, *p* = .035, $\eta_{p}^{2}$ = .078. Similarly for all groups there was a significant reduction in latency to the peak amplitude as shown by Fig. 1C, row 4, CR latency to peak, and results of within-subjects contrasts for blocks 1,2,3,5: BLK 1 v 8, *F*(1, 55) = 44.16, *p* < .001, $\eta_{p}^{2}$ = .434; BLK 2 v 8, *F*(1, 55) = 8.79, *p* = .005, $\eta_{p}^{2}$ = .137; BLK 3 v 8, *F*(1, 55) = 12.32, *p* = .001, $\eta_{p}^{2}$ = .183; BLK 5 v 8, *F*(1, 55) = 4.14, *p* = .047, $\eta_{p}^{2}$ = .070.

Analysis of responses in the late time window showed that as trace conditioning proceeded there was an increase in onset and peak amplitude as CRs to delay conditioning extinguished and true CRs to trace were learned. The fall in number of CRs during trace conditioning related to the extinction of early, generalised responses.

### 3.5 Trial rejection

Nicolson et al. (2002) showed significant differences in alpha responding between dyslexic and typical groups of adults, which indicates an increase in sensitivity to a tone CS. We therefore looked at alpha responses, responses made in the baseline period and the total number of trials rejected. The AM, LM and SLI groups did not differ in any of these measures in either delay or trace conditioning. All data failed the Shapiro–Wilk test of normality so Kruskal–Wallis chi-squared test was used to determine levels of significance between the groups. However, in no case did the chi-squared values approach statistical significance.

### 3.6 Psychometric tests and conditioning

We tested the ideas put forward by Ullman (2004) that “the cerebellum is expected to be involved in the search of lexical items, and possibly in the error-based learning of the rules that underlie the regularities of complex structures” through the proposed role in phonological working memory and influence upon Broca’s region (Desmond and Fiez, 1998). We predicted that delay conditioning would correlate with the children’s (SLI + LM) ability in grammar as measured by TROG together with the children’s ability in phonological processing as tested by the NEPSY non-word repetition. No significant correlation was found (see Table S2 for full analysis).

We also tested the idea put forward by Ullman that the acquisition of vocabulary engages in the main the declarative memory system including the hippocampal formation. Thus we predicted that we should find a relationship between trace conditioning and the children’s (SLI + LM) receptive and expressive vocabulary as measured by BPVS and Picture Naming. No significant correlation was found (see Table S2).

## 4 Discussion

The main finding of this study is that children with SLI and age and language-matched typical developing children did not differ in learning to delay and trace conditioning. These results are in agreement with a recent study by Steinmetz and Rice (2010) in which delay conditioning was investigated in children with SLI. In that study the US was presented to the left eye thus engaging mainly the left cerebellum in conditioning. One premise for our current study was that the right cerebellum is engaged more than the left in language, so we presented the US to the right eye thereby engaging the right cerebellum. Together with Steinmetz and Rice (2010) our results show that brain regions normally engaged in Pavlovian delay conditioning of the eyeblink response, particularly those within the cerebellum, are not impaired in in children with SLI. In addition we have shown that structures normally engaged in trace eyeblink conditioning, within the right cerebellum, medial temporal lobe and neocortex, function normally in children with SLI.

### 4.1 Generalisation and extinction

In order to prevent confounding of learning to trace after conditioning to delay we kept the CS duration constant and used a different ISI for delay and trace conditioning. The difference in parameters revealed considerable generalisation from the tone CS used in delay to the white noise CS used in trace conditioning in all groups. These results are similar to that shown in a recent study of the effects of foetal alcohol syndrome on delay and trace eyeblink conditioning. Jacobson et al. (2011) showed retention of delay conditioning in their typical children aged 11 years, even after an intervening gap of 0.4–1.8 years. In that study early responses matched to the ISI used in delay conditioning showed significant extinction after 50 trials of trace conditioning. Participants in our study show a similar extinction of delay conditioning.

### 4.2 Consolidation

A few children showed very poor learning during delay conditioning but all immediately showed early responses in the second session. Learning had continued to develop without the presence of any stimuli in all groups of children. This result is different to that shown by children with foetal alcohol syndrome (FAS). Children with FAS who fail to learn to delay conditioning also fail to learn to trace conditioning (Jacobson et al., 2011). In our study, even those children who gave few CRs during delay conditioning showed consolidation and generalisation at the start of trace conditioning indicating normal cerebellar function.

### 4.3 Effects of reading ability

We distinguished between good and poor readers because studies of eyeblink conditioning in dyslexics had found impairments ranging from a failure in timing of the CR in young adults (Nicolson et al., 2002: range 13–24 yrs) to almost no conditioning in children (Coffin, Baroody, Schneider, & O’Neill, 2005: range 9–10 yrs). In our study the LM group ranged from 4–7 years, AM and SLI ranged 7–11 years. We found no evidence for poorer learning in children with SLI who were poor readers. All children with SLI were unimpaired in delay and trace conditioning.

### 4.4 Discrepancies with previous literature

The results of Nicolson et al. (2002) and Coffin, Baroody, Schneider, and O’Neill (2005) have added to the idea that a deficit in right cerebellar function is a major factor in dyslexia (Pernet, Poline, Demonet & Rousselet, 2009). Evidence provided by Coffin et al. (2005) was particularly compelling, since in their study children with dyslexia gave very few CRs, whereas in the Nicolson et al., study the deficit was in CR timing. One difference, apart from age, between the study of Coffin art al. and Nicolson et al. was the ISI. The shorter ISI used by Coffin et al., (400 vs 720 ms) may have been suboptimal for conditioning in children with dyslexia. Thus it is particularly surprising that our SLI poor reader group showed no impairment even though we used the same ISI as Coffin et al. Our result is consistent with a recent study in which no impairment of delay conditioning of the right eye was found in adults with dyslexia even though an ISI of 700 ms was used (Laasonen et al., 2012).

Laasonen et al., however, found significantly poorer learning in their dyslexic group of adults during trace conditioning, whereas in our study children with SLI who were poor readers responded to trace conditioning the same as typical children. One difference, apart from age, between our study and that of Laasonen et al., was the relationship between ISI and CS duration. In Laasonen et al., the trace interval was similar to ours (600 vs 500 ms) but the ISI was shorter (700 vs 1000 ms). Thus the CS duration was much shorter than in our study (100 vs 500 ms) which may be suboptimal for trace conditioning in dyslexics.

### 4.5 A procedural memory deficit – striatum or cerebellum?

Ullman and Pierpont (2005) argued that SLI is a consequence of a deficit in procedural memory based on the cortico-striatal system. Nicolson and Fawcett (1990, 2007, 2011) argued that dyslexia is due to a deficit in procedural memory based on the cortico-cerebellar system. Both arguments are based upon the idea of a working memory system described by Baddeley (1992). Many children with SLI are extremely poor at non-word repetition due in part to poor phonological memory (Gathercole & Baddeley, 1990). Many children with dyslexia are also poor at non-word repetition (Rack, Snowling, & Olson, 1992). One might expect that the mechanism underlying poor nonword repetition and its locus to be the same in both SLI and dyslexia. But although the mechanism may be the same, the locus may be within the different cortical pathways suggested above. Also, a deficit may occur anywhere between the input and output of the phonological memory system (Snowling, Chiat, & Hulme, 1991) and so the deficit in SLI may be in a different phase of phonological memory than in dyslexia but within the same cortical pathway. Given the deficits in eyeblink conditioning shown by Nicolson et al. (2002) and Coffin et al. (2005) we expected to have been able to distinguish between these possibilities. We tested children with SLI, some of whom had poor reading abilities and only those children were poor at nonword repetition. If the pathway underlying poor nonword repetition involved the cerebellum we might have found a deficit in eyeblink conditioning only in those children but we found none.

In a sample of children who overlapped substantially with those in the current study, Hsu & Bishop (2013) found impaired performance on one nonverbal test of implicit learning, the serial reaction time task, but not in another, the pursuit rotor task. Our null result provides further evidence that procedural learning deficits in SLI are not general and helps narrow the mechanisms that are compromised. However, the mechanism by which implicit serial reaction time task fails in SLI and dyslexia (Menghini, Hagberg, Caltagirone, Petrosini, & Vicari, 2006) is currently unknown as both cerebellum, particularly lobule HVI, and basal ganglia are essential components (Bermard & Seidler, 2013; Doyon et al., 2009).

### 4.6 What does eyeblink conditioning reveal?

The involvement of the cerebellum in SLI was postulated because of its proposed role in verbal working memory (Marvel & Desmond, 2010). We argued that eyeblink conditioning will test the integrity of the same parts of the cerebellum that are thought to be involved in language and reading through a role in verbal working memory. If true, then together with Steinmetz and Rice (2010) and Laasonen et al. (2012), we have provided evidence that those regions of the cerebellum are functioning normally in children with language and reading difficulties. The simplest explanation is that impairment of cerebellar function does not form part of the neurobiology of SLI.

Closure of the eyelids involves activation of microzones mainly within lobule HVI projecting to the anterior interpositus nucleus, then via the red nucleus and dorsolateral facial nucleus to the orbicularis oculi muscle (Gonzalez-Joekes & Schreurs, 2012; Hesslow, 1994). The facial nucleus also controls the orbicularis oris and other muscles involved in speech. But microzones that control the articulators and are engaged in the working memory model of Desmond and colleagues might be separate from those controlling eyeblinks. For example microzones within HVI receiving information from and having some control over the jaw, tongue and pharynx from the mandibular branch of the trigeminal nerve and hypoglossal and glossopharyngeal nerves within the neck may not be engaged in eyeblink conditioning. Durisko and Fiez (2010) provided evidence that different regions within lobule HVI can have different functions in working memory and it is known that eyeblink related microzones are highly localised within lobule HVI in rabbits (Mostofi et al., 2010). We cannot rule out a focal cerebellar impairment in SLI, but together with Steinmetz and Rice (2010) and Laasonen et al. (2012), we have provided evidence that the general cerebellar deficit underlying dyslexia as proposed by Nicolson and Fawcett (1990, 2007) is false. If the cerebellum is involved in language and reading impairment it must be through a mechanism that engages extremely specific regions within the cortex and deep nuclei.

## Figures and Tables

**Table 1:** Participants: numbers, age and psychometric test scores.

Group	SLI_TR		SLI_PR		LM		AM		SLI_TR		SLI_PR		AM	

Delay conditioning (n)	17		22		21		21							
Trace conditioning (n)	13		19		13		20							

*Age*														
Range (yrs)	6.5–11.3		6.8–11.1		4.8–7.4		7.2–10.8							
M, SD (yrs)	8.7	1.4	9	1.3	6.1	0.7	8.5	1.2						

	Raw scores								Percent accuracy					
	M	SD	M	SD	M	SD	M	SD	M	SD	M	SD	M	SD

RCPM	103.4	11.8	99.4	11.7	111.9	10.1	105.5	8.7						
NEPSY NWR	28.3	7.9	20.9	7	28.1	8	32.1	5	94.1	15	79.8^*^	11.6	102.6	9.7
BPVS	71.6_a_	12.6	73.4_a_	20.2	73.4_a_	14.7	–	–	87.8	9.8	89.9	8.4	–	–
Picture naming	11.4_a_	4.1	11.4_a_	3.2	11.5_a_	3.4	–	–	82.9^*^	11.5	81.6^*^	11.7	–	–
Syntactic formulation	19.6	5.5	16.2	6.2	21.1	5.2	–	–	82.4^*^	8.5	81.4^*^	13	–	–
TROG	9.9_bc_	3.4	8.5_b_	4	11.2_c_	2.9	14.8_d_	2.2	77.5^*^	14.3	71.9^*^	12.4	100.1	8.6

*ERRNI*														
Initial story	17.5	5.4	19.7	7.4	16.7	5.0			84.3	11.5	93.2	13.6	–	–
Recall story	12.4	6.4	16.0	9.6	12.9	7.1			84.3	14.0	94.2	19.8	–	–
Comp	8.9	3.5	10.3	3.4	9.3	2.8	–	–	83.6^*^	16.9	88.2	15.9	–	–
*TOWRE*														
SWR	52.5	17	28.6	11.6	29.4	20.6	53.9	15.2	101.1	10.6	77.6^*^	6.7	104.2	10.1
PDEC	23	13.1	7.8	5.3	13.9	12.4	24.5	12	98.8	12.6	77.7^*^	7.6	100.5	7.9

For scaled scores: ^*^ denotes greater than 1SD below norm. Means in the same row that do not share subscripts differ at *p* < .05 in ANOVA (subscript a) or the Turkey HSD post hoc test (subscript b, c and d). *General non-verbal intelligence*: RCPM, (Raven, Court & Raven, 1986). Phonological processing: NEPSY, (Korkman, Kirk, & Kemp, 1998), raw/46; *Receptive vocabulary*: BPVS-2, (Dunn, Dunn, Whetton, & Burley, 1997), raw/168. *Expressive vocabulary*: Picture naming subtest of ACE 6–11 (Adams, Cooke, Crutchley, Hesketh, & Reeves, 2001), raw/25. *Expressive syntax*: Syntactic formulation subtest of ACE 6–11, raw/32. *Receptive grammar*: TROG-2, (electronic version; Bishop, 2003). *Comprehension*: ERRNI (Bishop, 2004), raw/18. *Reading ability*: TOWRE (Torgesen, Wagner, & Rashotte, 1999): SWR = sight word reading efficiency, raw/104. PDEC = phonemic decoding efficiency, raw/63.

**Table 2:** ANOVA for Session 1 and 2 with between-subject factor group (GRP) and within-subject factor block (BLK).

Measure	Source	Session 1				Session 2							
		Delay				Generalised delay				Trace			
						Early time window				Late time window			
		*df*	*F*	*p*	$\eta_{p}^{2}$	*df*	*F*	*p*	$\eta_{p}^{2}$	*df*	*F*	*p*	$\eta_{p}^{2}$
CR number	GRP	3	3.09	.912	.007	3	.296	.828	.014	3	.33	.805	.016
	Error	77	(2.21)			61	(2.56)			61	(2.74)		
	BLK	5.46	12.53⁎⁎	<.001	.140	5.80	13.01⁎⁎	<.001	.176	7	3.18⁎⁎	.003	.049
	BLK ^*^ GRP	16.38	0.83	.655	.031	17.41	0.88	.598	.042	21	0.97	.495	.046
	Error	420.47	(3.16)			353.93	(3.44)			427	(3.18)		

CR amplitude	GRP	3	1.417	.244	.052	3	1.17	.328	.055	3	1.01	.394	.047
	Error	77	(1053.40)			61	(1460.07)			61	(1188.38)		
	BLK	5.13	2.772⁎	.017	.035	4.21	21.30⁎⁎	<.001	.259	4.99	2.01	.077	.032
	BLK ^*^ GRP	15.38	0.859	.613	.032	12.63	1.44	.142	.066	14.97	1.33	.183	.061
	Error	394.76	(1496.56)			256.87	(1380.89)			304.37	(1439.95)		

CR latency to onset	GRP	3	.973	.416	.073	3	.36	.785	.019	3	.70	.555	.037
	Error	37	(1015.16)			56	(4365.57)			55	(803.09)		
	BLK	4.99	1.696	.138	.044	5.61	4.53⁎⁎	<.001	.075	7	6.90⁎⁎	<.001	.112
	BLK ^*^ GRP	14.98	0.900	.565	.068	16.83	1.11	.344	.056	21	0.90	.591	.047
	Error	184.76	(5082.24)			314.20	(6545.20)			385	(2652.46)		

CR latency to peak	GRP	3	1.191	.326	.088	3	.088	.987	.005	3	0.65	.589	.034
	Error	37	(1596.83)			57	(4408.29)			55	(1072.98)		
	BLK	4.74	3.551⁎⁎	.005	.088	5.56	2.79⁎	.014	.047	5.62	7.43⁎⁎	<.001	.119
	BLK ^*^ GRP	14.22	0.806	.663	.061	16.98	0.65	.849	.033	16.85	0.36	.991	.019
	Error	175.38	(5183.72)			316.99	(8584.65)			308.99	(3640.22)		

UR amplitude	GRP	3	.310	.818	.014					3	1.26	.297	.058
	Error	66	(9377.68)							61	(2273.70)		
	BLK	3.36	3.236⁎	.019	.047					4.09	8.11⁎⁎	<.001	.117
	BLK ^*^ GRP	10.08	1.186	.301	.051					112.27	0.97	.475	.046
	Error	221.71	(4122.42)							249.61	(2107.55)		

UR latency to peak	GRP	3	1.046	.378	.045					3	.41	.746	.020
	Error	66	(946.38)							59	(622.76)		
	BLK	5.80	2.887⁎	.010	.042					7	0.40	.901	.007
	BLK ^*^ GRP	17.41	0.933	.536	.041					21	0.89	.604	.043
	Error	382.99	(1590.07)							413	(1177.62)		

*Note*. Values in parentheses represent mean square errors.

⁎ *p* < .05.

⁎⁎ *p* < .01.

## Appendix A Supplementary material

Supplementary data 1 Summary of delay and trace conditioning. Group means and standard deviations of responses in 80 paired trials.
