# Supplementary material for: Children with Specific Language Impairment are not impaired in the acquisition and retention of Pavlovian delay and trace conditioning of the eyeblink response
Source: Brain Lang. 2013 Dec;127(3):428–39. doi: 10.1016/j.bandl.2013.08.001 (PMC3847270; doi:10.1016/j.bandl.2013.08.001)
Supplement: Supplementary data 1 — Summary of delay and trace conditioning. Group means and standard deviations of responses in 80 paired trials. [file mmc1.docx]

**Table S1**. Summary of delay and trace conditioning. Group means and standard deviations of responses in 80 paired trials.

|  | Group | Session 1 | | Session 2 | | | |
| --- | --- | --- | --- | --- | --- | --- | --- |
|  |  | Measure | | Generalised Delay | | Trace | |
|  |  |  | | Early Time Window | | Late Time Window | |
|  |  | M | SD | M | SD | M | SD |
| CR  % | AM | 27.92 | 10.85 | 55.13 | 17.18 | 48.06 | 17.79 |
|  | LM | 26.53 | 13.67 | 55.48 | 16.04 | 53.37 | 15.84 |
|  | SLI_TR | 30.00 | 16.23 | 51.35 | 13.12 | 48.75 | 14.85 |
|  | SLI_PR | 27.84 | 17.66 | 56.64 | 17.11 | 51.25 | 18.39 |
| CR  Amplitude | AM | 59.80 | 32.42 | 76.40 | 30.87 | 65.42 | 27.31 |
|  | LM | 43.34 | 34.76 | 96.63 | 45.51 | 86.22 | 41.62 |
|  | SLI_TR | 50.33 | 36.89 | 111.73 | 125.95 | 96.61 | 68.68 |
|  | SLI_PR | 40.79 | 24.99 | 73.21 | 38.22 | 69.61 | 39.36 |
| CR  Latency  To Onset | AM | 259.77 | 35.86 | 282.84 | 55.16 | 749.06 | 52.62 |
|  | LM | 250.86 | 33.74 | 304.56 | 75.23 | 741.79 | 48.32 |
|  | SLI_TR | 268.85 | 21.46 | 288.99 | 83.47 | 764.20 | 28.52 |
|  | SLI_PR | 261.96 | 41.86 | 284.61 | 49.86 | 753.19 | 28.91 |
| CR  Latency  To Peak | AM | 308.10 | 41.01 | 409.69 | 64.93 | 824.92 | 65.98 |
|  | LM | 296.12 | 48.17 | 413.05 | 63.79 | 817.79 | 65.43 |
|  | SLI_TR | 319.57 | 39.58 | 424.85 | 73.94 | 846.58 | 35.22 |
|  | SLI_PR | 306.61 | 43.93 | 419.24 | 60.27 | 840.91 | 31.27 |
| UR  Amplitude | AM | 142.94 | 79.28 |  |  | 115.48 | 51.72 |
|  | LM | 116.69 | 68.34 |  |  | 136.02 | 46.01 |
|  | SLI_TR | 144.50 | 132.98 |  |  | 117.10 | 45.10 |
|  | SLI_PR | 133.75 | 91.35 |  |  | 102.74 | 44.34 |
| UR  Latency  To Peak | AM | 555.00 | 26.17 |  |  | 1138.46 | 22.28 |
|  | LM | 558.50 | 31.95 |  |  | 1147.85 | 18.51 |
|  | SLI_TR | 568.57 | 42.14 |  |  | 1141.15 | 32.64 |
|  | SLI_PR | 551.22 | 43.60 |  |  | 1140.14 | 24.90 |

Table S2. Linear correlation between psychometric test and eyeblink conditioning in SLI_GR, SLI_PR and LM groups.

| DELAY | N=60 | Num | Amp | LatOns | LatPk |  |  |  |  |
| --- | --- | --- | --- | --- | --- | --- | --- | --- | --- |
| TROG | COR | 0.08 | 0.11 | -0.01 | 0.02 |  |  |  |  |
|  | SIG | 0.55 | 0.41 | 0.96 | 0.91 |  |  |  |  |
| NWR | COR | 0.11 | 0.00 | 0.02 | 0.07 |  |  |  |  |
|  | SIG | 0.41 | 0.99 | 0.86 | 0.61 |  |  |  |  |
| TRACE | N=45 | NumL | AmpL | LatOnsL | LatPkL |  |  |  |  |
| BPVS | COR | -0.07 | -0.15 | 0.15 | 0.07 |  |  |  |  |
|  | SIG | 0.64 | 0.31 | 0.34 | 0.66 |  |  |  |  |
| PicNam | COR | -0.02 | -0.23 | 0.11 | 0.09 |  |  |  |  |
|  | SIG | 0.90 | 0.13 | 0.48 | 0.57 |  |  |  |  |

_Trace conditioning responses in late time window. Values are Pearson correlation._
